# 3D printing of a bio-based ink made of cross-linked cellulose nanofibrils with various metal cations

**DOI:** 10.1038/s41598-021-85865-4

**Published:** 2021-03-19

**Authors:** J. Benedikt Mietner, Xuehe Jiang, Ulrica Edlund, Bodo Saake, Julien R. G. Navarro

**Affiliations:** 1 Institute of Wood Science, Universität Hamburg, Hamburg, Germany; 2 Fiber and Polymer Technology, KTH Royal Institute of Technology, Teknikringen 56, 100 44 Stockholm, Sweden

**Keywords:** Bioinspired materials, Materials chemistry

## Abstract

In this work, we present an approach to cross-link cellulose nanofibrils (CNFs) with various metallic cations (Fe^3+^, Al^3+^, Ca^2+^, and Mg^2+^) to produce inks suitable for three-dimensional (3D) printing application. The printability of each hydrogel ink was evaluated, and several parameters such as the optimal ratio of M^n+^:TOCNF:H_2_O were discussed. CNF suspensions were produced by mechanical disintegration of cellulose pulp with a microfluidizer and then oxidized with 2,2,6,6-tetramethylpiperidine-1-oxyl (TEMPO). Finally, metal cations were introduced to the deprotonated TEMPO-oxidized CNF (TOCNF) suspension to cross-link the nanofibrils and form the corresponding hydrogels. The performances of each gel-ink were evaluated by rheological measurements and 3D printing. Only the gels incorporated with divalent cations Ca^2+^ and Mg^2+^ were suitable for 3D printing. The 3D printed structures were freeze-dried and characterized with Fourier transform infrared spectroscopy (FT-IR) and Scanning Electron Microscopy (SEM). The better interaction of the TOCNFs with the divalent metallic cations in terms of printability, the viscoelastic properties of the inks, and the variation trends owing to various metal cations and ratios are discussed.

## Introduction

The need to replace petroleum-based products with biodegradable and renewable resources, to produce high-performance functional materials is one of the greatest challenges for a future sustainable society. For this purpose, bio-based polymers have attracted considerable attention over the past decades. Cellulose, and its derivatives, fulfill those needs by offering many advantages such as renewability, biodegradability, and to some extent recyclability^1–3^. Cellulose nanofibrils (CNF) is a highly promising candidate for a wide panel of applications, ranging from composites, water purification to drug delivery^4–7^. CNF can be extracted from numerous lignocellulosic source materials through mechanical disintegration^8^. Typical CNF dimensions comprise widths between 5 and 20 nm and a wide range of lengths, typically several micrometers^9^.

Recently, several groups demonstrated that CNF could be structured through 3D printing processes^10–16^. The demonstrated 3D printed CNF objects showed great potential as 3D printed tablets for controlled drug release and as 3D printed bioactive composites in tissue engineering and wound dressing applications. Markstedt et al.^17^ produced a biobased ink made of cross-linked CNF, alginate, and CaCl_2_. The CNF-alginate cross-linked structure proved to be a viable scaffold for hosting human nasoseptal chondrocyte cells. Leppiniemi et al.^18^ developed a 3D ink based on alginate, avidin protein-modified CNF and glycerin. In this study Leppiniemi et al. were using CNF as a strengthening additive and CaCl_2_ as a cross-linker, what leads to a significantly more stable shape fidelity after 3D printing. Moreover, the 3D printed object showed good tissue compatibility and great potential in biomedical applications such as in wound dressings. Li et al.^19^ produced a 3D printed structure made of nanocellulose and carbon nanotubes. The object was first 3D printed and cross-linked with CaCl_2_ later on. Several drying protocols were applied to the printed hydrogel and freeze-drying was found to be the most efficient strategy.

CNF has an abundance of hydroxyl groups on the fibril surfaces which lead to strong hydrogen interaction (i) between fibrils (inter-fibrils interaction that leads to fibril agglomeration) and (ii) with water molecules, endowing viscoelasticity and shear thinning properties that are advantageous for 3D printing^20^. The viscoelastic properties help to maintain the structural shape integrity of the CNF structure after complete removal of water from a CNF hydrogel upon freeze-drying, under appropriate conditions^14^. To enhance and enlarge the application range of those nanofibrils and increase the compatibility and adhesion to other matrices (such as hydrophobic thermoplastics), several surface modification chemistries were explored^21,22^. Among them, the catalytic oxidation with 2,2,6,6-tetramethylpiperidine-1-oxyl (TEMPO) proved to be an efficient method for the modification of CNF hydroxyl groups^23^. One advantage of the TEMPO-mediated oxidation is that the reaction can be carried out in water and under mild conditions. The resulting TEMPO-mediated oxidized CNF (TOCNF) has a high anionic charge density on the fibril surfaces. TOCNF suspensions behave like gels under moderate concentrations, however, they cannot withstand a high shear rate as the gel is easily disrupted^24^. As previously reported, stable TOCNF gels can be obtained through the cross-linking of the CNF carboxylate groups with various polymers and/or divalent and trivalent metal cations^10,24–31^. The cross-linking process can strengthen the network structures of the oxidized CNF-based hydrogels.

Our final goal is to develop 3D printable CNF hydrogel inks by cross-linking TEMPO-oxidized CNF with divalent and trivalent metal cations. The properties and 3D printing performance of the cross-linked TOCNF-based hydrogels were studied and evaluated and the ability of different metal ions to serve as stabilizing cross-linkers was assessed. In this study, our primary aim was to investigate and correlate the mechanical properties of the cross-linked CNF hydrogels with their ability to be later-on process through 3D printing. In this paper, we highlight the importance of choosing the correct metal cation (di- or trivalent), the ratio of the different precursors (M^n+^:TOCNF:H_2_O ratios ranging from 1:1:10 to 1:1:25), and the solid content of the cross-linked CNF gels. Those parameters drastically affect the mechanical properties of the hydrogel. As an example, the choice of a wrong balance between those parameters can yield either a too weak, liquid CNF suspension or a too stiff, dense hydrogel, and therefore produce an unprintable bio-ink. This article reviews the optimal conditions for printing a bio-based gel made of cross-link CNF.

## Results and discussion

**Figure 1:**
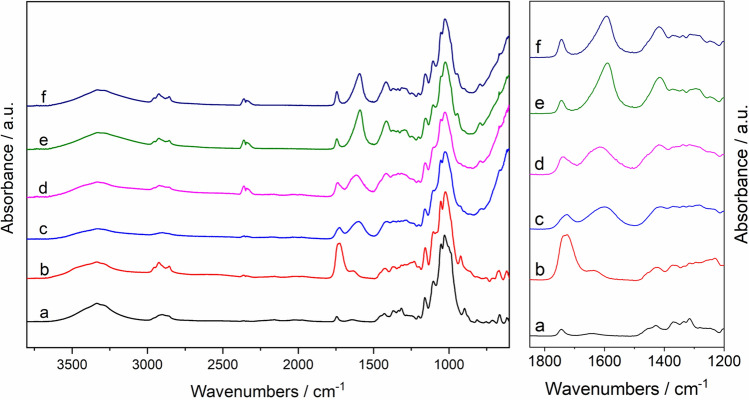
ATR-FTIR spectra of the dried TOCNF suspension and TOCNF–M^n+^ hydrogels: (a) pristine CNF (b) TOCNF, (c) TOCNF–Fe^3+^, (d) TOCNF–Al^3+^, (e) TOCNF–Ca^2+^, (f) TOCNF–Mg^2+^. Left: full spectra, right: region of interest.

Cross-linked hydrogels were prepared from TEMPO-oxidized CNF with various metallic cations (Fe^3+^, Al^3+^, Ca^2+^, and Mg^2+^). Gelation of the TOCNF suspension occurred immediately upon the addition of the metal cation solution, through diffusion of the metal cations into the deprotonated TOCNF dispersion followed by electrostatic interactions between the metal cations and the negative charge of the TOCNF carboxylate groups. All the hydrogels (TOCNF–M^n+^) were left undisturbed overnight to enable thorough diffusion of cations into the preformed gels. All gels prepared through this method were macroscopically homogeneous and were slightly less transparent than the TOCNF starting dispersions. The Fe^3+^ cross-linked TOCNF gels (TOCNF–Fe^3+^) were yellow (which is typical for this ion complex) while the TOCNF gels cross-linked with Al^3+^ (TOCNF–Al^3+^), Ca^2+^ (TOCNF–Ca^2+^) and Mg^2+^ (TOCNF–Mg^2+^) remained colorless. The cross-linked gels were characterized with ATR-FTIR spectroscopy (Fig. 1).

As shown in Fig. 1, the spectrum of the initial and unmodified CNF exhibits the characteristic bands of the nanocellulose with bands localized at 3335 cm^−1^ (ʋ_OH_), 2905 cm^−1^ and 2860 cm^−1^ (ʋ_C-H_), 1637 cm^−1^ (δ_OH_), 1429 cm^−1^ (δ_CH2_), 1369 cm^−1^ (δ_C–H_) and 1335 cm^−1^ (δ_O–H_). In addition to those characteristic bands, the TOCNF spectrum shows a strong additional absorption band localized at 1725 cm^−1^ which is attributed to the vibration of the carbonyl bond (ʋ_C=O_) in the carboxylic group. The presence of this new band confirms the successful chemical conversion of CNF into TOCNF. After cross-linking of the TOCNF with a metal cation, new bands appear in the region 1650–1400 cm^−1^.

Vibration assignments for the most relevant bands are listed in Table 1. With or without cross-linking, the broad bands localized between 3297 and 3335 cm^−1^ (ʋOH stretching vibrations) remain unchanged.

**Table 1:** IR Wavenumbers for CNF, TOCNF, and TOCNF–M^n+^ hydrogels.

Samples	ʋ_OH (H-bonded)_/cm^−1^	ʋ_C=O_/cm^−1^	ʋ_as, OCO_/cm^−1^	ʋ_s, OCO_/cm^−1^
CNF	3335	–	–	–
TOCNF	3334	1725	–	–
CNF-Fe^3+^	3326	1725	1601	1412
CNF-Al^3+^	3330	1735	1617	1419
CNF-Ca^2+^	3330	1744	1590	1418
CNF-Mg^2+^	3330	1744	1593	1418

As mentioned earlier, the TOCNF spectrum exhibits a strong additional absorption band localized at 1725 cm^−1^ (ʋ_C=O_). A shift of this carbonyl band is observed after metal ion cross-linking of the TOCNF. The bands for these vibrations in TOCNF–M^n+^ spectra are attributed to un-complexed carboxylate groups that still exist in the carboxylic acid form. Thus, the divalent cations Ca^2+^ and Mg^2+^ incorporated better with deprotonated TOCNF than the trivalent cations Fe^3+^ and Al^3+^, due to the relatively stronger ʋ_s, OCO_ stretching vibration of TOCNF–Ca^2+^ and TOCNF–Mg^2+^, whereas the hydrogels with trivalent cations Fe^3+^ and Al^3+^ had less incorporation and more un-complexed C=O groups that exist as carboxylic acid form^24^.

In the TOCNF–M^n+^ spectra, the symmetric and asymmetric bands (ʋ_as/s, OCO_) are also shifted towards lower wavenumbers, which is due to the formation of ionic bonds between the cations and the carboxylate groups of the surface-modified cellulose^32^.

Various cations and various M^2+^:TOCNF:H_2_O ratios were investigated (Table 2). The gelation process was faster with the addition of trivalent cations (Fe^3+^, Al^3+^) than with divalent cations (Ca^2+^, Mg^2+^). Lower yields were observed when divalent ions were used for the cross-linking. The amounts of TOCNF–Fe^3+^ and TOCNF–Al^3+^ gels were similar and about twice as high as the yields of TOCNF–Ca^2+^ and TOCNF–Mg^2+^.

**Table 2:** Solid contents of 3D printed TOCNF–Ca^2+^ and TOCNF–Mg^2+^ hydrogels at various ratios of M^2+^:TOCNF:H_2_O.

M^2+^:TOCNF:H_2_O	TOCNF–Ca^2+^ wt%	TOCNF–Mg^2+^ wt%
1:1:1	1.67	1.17
1:1:10	1.03	0.79
1:1:20	0.52	0.95
1:1:25	1.39	0.92
1.5:1:25	1.19	1.00

Hydrogels synthesized with a M^n+^:TOCNF ratio of 1:1 and without water dilution did not exhibit any fluidity and were mechanically too robust and rigid to pass through the 3D printer nozzle and could therefore not be 3D printed. The same was observed for M^n+^:TOCNF gels with a M^n+^:TOCNF:H_2_O ratio of 1:1:1, regardless of the valency of the cation.

To obtain 3D printable gels, the hydrogels were swollen through the addition of water. The addition of water during gel preparation (M^n+^:TOCNF:H_2_O ratios ranging from 1:1:10 to 1:1:25) influenced the rheological behavior of the gels and the rigidity decreased in the order TOCNF–Fe^3+^  >  TOCNF–Al^3+^ >  TOCNF–Ca^2+^  >  TOCNF–Mg^2+^ (Fig. 4). The synthesized hydrogels obtained with trivalent cations were unprintable regardless of the M^n+^:TOCNF:H_2_O ratio, probably due to the high gel density that does not meet the specific rheological requirements (for example shear thinning) and therefore could not be pneumatically extruded since they blocked the 3D printer nozzle.

On the contrary, hydrogels cross-linked with divalent cations could be 3D printed, however, the printed objects became inhomogeneous (heavy structural defects) at M^n+^:TOCNF:H_2_O ratios ranging from 1:1:10 to 1:1:20. When the ratio was 1:1:25, the gels of TOCNF–Ca^2+^ offer the best printing performance while the TOCNF–Mg^2+^ hydrogel was not firm enough for shape retention when printed in the form of a cube. When the ratio was kept at 1.5:1:25, the TOCNF–Ca^2+^-gel was inhomogeneously printed and the TOCNF–Mg^2+^-gel was still too fluid, although this would be the perfect ratio according to the Derjaguin–Landau–Verwey–Overbeek [DLVO (This theory explains that chemical factors, such as pH and electrolyte concentration, can reduce the thickness of the electrical bilayers of colloids and cause an aggregation of colloids through Brownian motion.)] theory, based on calculations from Fukuzumi et al.^33^ in a study on the dispersion stability and aggregation behavior of TEMPO-oxidized cellulose nanofibrils in water as a function of salt addition.

The initial TOCNF suspension is opaque, nearly transparent and very fluidic. The direct 3D printing of the initial TOCNF suspension in a bath what contains the metal cation solution for post-printing cross-linking was investigated, but was unfortunately not successful.

**Figure 2:**
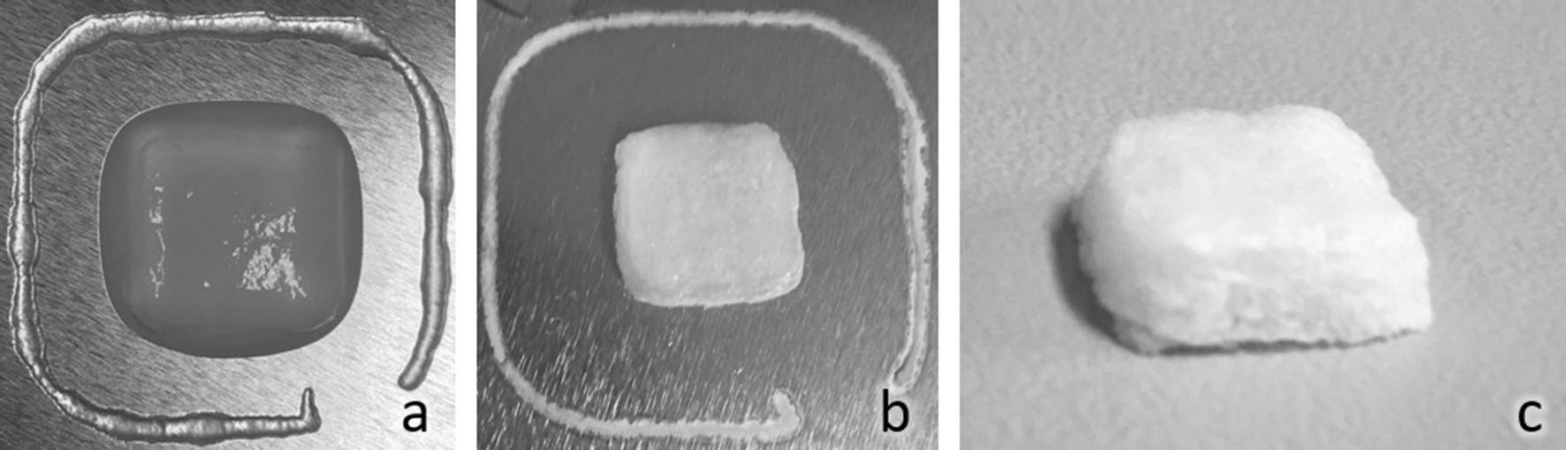
Representative images of printed TOCNF–Mg^2+^-gel (1:1:25, 2 × G). Gel cube (**a**) (10 × 10 × 5 mm) after printing in wet state, and (**b**, **c**) after freeze-drying.

Centrifugation with higher rotation speed (Table 3) had no significant impact on the performance of TOCNF–Ca^2+^ (solid content remained unchanged: 1.39 wt%) but affected the TOCNF–Mg^2+^ hydrogel with an increase of the solid content to 1.39 wt%. With this centrifugation step, TOCNF–Mg^2+^ hydrogels could be 3D printed as a cube with a good shape fidelity. After the freeze-drying process, the 3D printed TOCNF–Mg^2+^ hydrogels maintained good structural integrity (Fig. 2).

**Table 3:** Solid contents of 3D printed TOCNF–Ca^2+^ and TOCNF–Mg^2+^ hydrogels at the ratio of 1:1:25 with various centrifugation intensities.

Centrifugation intensities	TOCNF–Ca^2+^ wt%	TOCNF–Mg^2+^ wt%
1 × G	1.39	0.92
2 × G	1.40	1.33
3 × G	–^a^	1.39

^a^Not measured, because no need for 3 × G at TOCNF–Ca^2+^.

**Figure 3:**
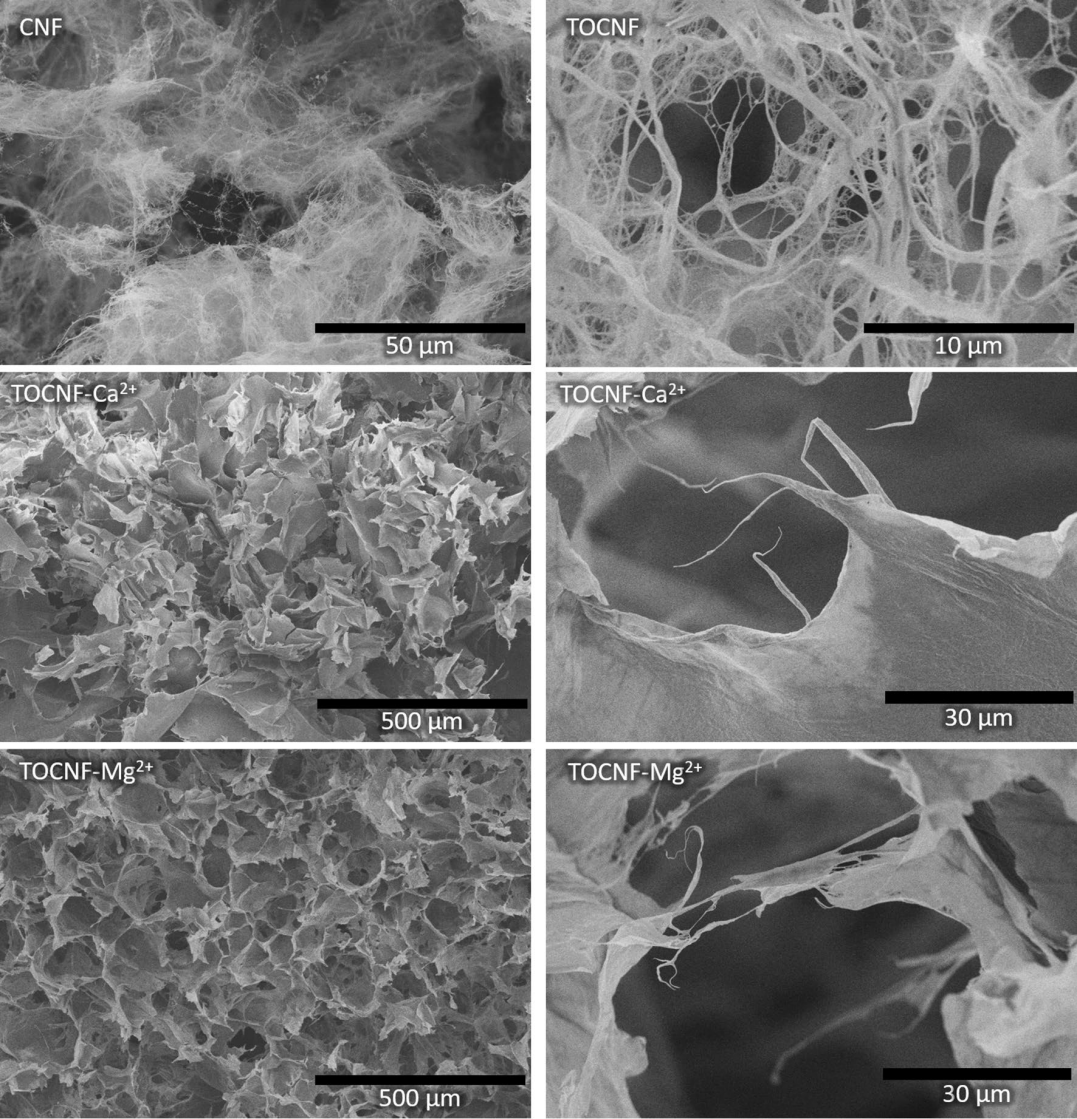
Representative SEM images of CNF, TOCNF, TOCNF–Ca^2+^ and TOCNF–Mg^2+^ at different magnifications.

The CNF, TOCNF, and the 3D printed and freeze-dried TOCNF–M^2+^ samples were also analyzed by Scanning Electron Microscopy (SEM) and the SEM images are shown in Fig. 3.

The SEM images of CNF and TOCNF in Fig. 3 show an agglomerated network of isolated fibrils. The images of the 3D printed and freeze-dried cubes of the cross-linked samples TOCNF–Ca^2+^ and TOCNF–Mg^2+^ show a highly porous structure with dense pore walls made from the cross-linked TOCNF. Cross-linking in combination with freeze-drying leads to a very high degree of interfibril interaction and the formation of dense sheets as observed in the TOCNF–Ca^2+^ sample (Fig. 3, middle right).

Tables 2 and 3 list the solid contents of the 3D printed TOCNF–Ca^2+^ and TOCNF–Mg^2+^ hydrogels after freeze-drying. The solid content of pristine TOCNF (2.64 wt%) decreases after the cross-linking process (TOCNF–M^2+^) probably due to the insufficient interaction between the metal cations and deprotonated TOCNF dispersions. Higher water contents within the TOCNF–Mg^2+^ (ratio 1:1:25) were decreased by more intensive centrifugation (Table 3), thus leading to a higher solid content and a better 3D printing performance of the resulting TOCNF–Mg^2+^ hydrogels.

**Figure 4:**
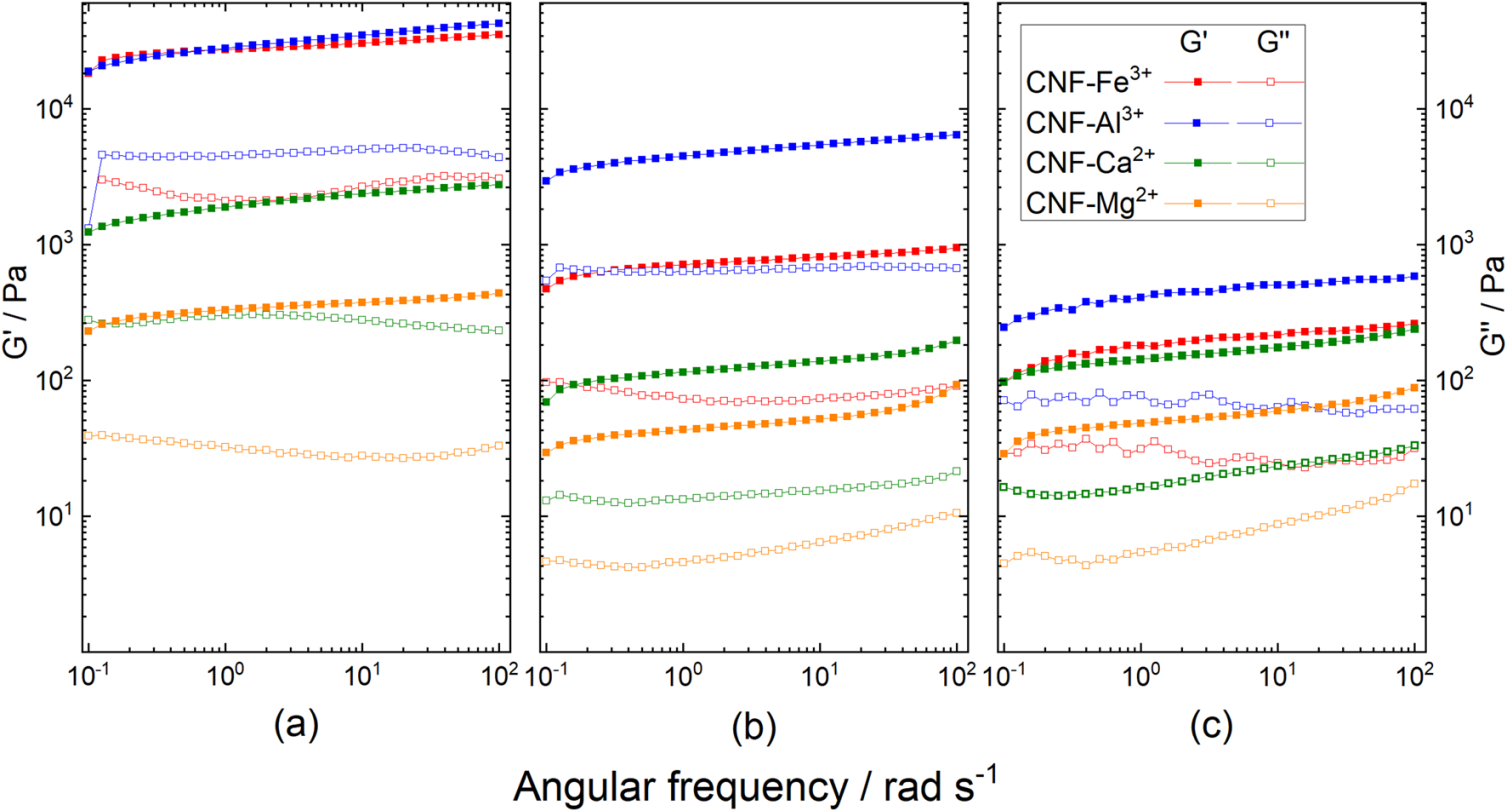
Viscoelastic properties of the TOCNF–M^n+^ hydrogels with various cations:Fe^3+^, Al^3+^, Ca^2+^, and Mg^2+^ are presented in red, blue, green, and orange, respectively. M^n+^:TOCNF(:H_2_O) ratios: (**a**) 1:1, (**b**) 1:1:25, and (**c**) 1.5:1:25. Storage modulus (G′) and loss modulus (G″) are symbolized with filled and open symbols, respectively.

Viscoelastic properties of the hydrogels, storage modulus (G′) and loss modulus (G″), are shown in Fig. 4. Moduli were measured as a function of a dynamic frequency sweep between 0.1 and 100 rad/s. The G′ values of the hydrogels are consistently larger than the G″ values in the entire angular frequency range. Moreover, both G′ and G″ values show similar small variations with frequency in the defined range, which indicate a stable gel state of TOCNF–M^n+^.

The dynamic moduli of TOCNF–Fe^3+^ and TOCNF–Al^3+^ are clearly higher than for TOCNF–Ca^2+^ and TOCNF–Mg^2+^, and they present a significant declining trend after dilution with water during the gelation process. The highest storage modulus of the hydrogels with the ratio of M^n+^:TOCNF at 1:1 (up to Gʹ = 40 kPa for Fe^3+^:TOCNF, 1:1) demonstrated the high rigidity and unprintability of those gels. Interestingly, increasing the proportion of metal cations to a M^n+^:TOCNF:H_2_O ratio of 1.5:1:25 decreased the dynamic modulus, if compared with the ratio of 1:1:25, at which TOCNF–Ca^2+^ had the best 3D printing performance. It is possibly due to more substantial intra-fibril interactions rather than an inter-fibril cross-linking, at higher amounts of metal cations and wider dispersed TOCNFs^34^. Additionally, the rheological measurements of hydrogels with M^n+^:TOCNF ratios of 1:1 and the TOCNF–Al^3+^ hydrogel (M^n+^:TOCNF:H_2_O ratio 1:1:25) present some deviations, and the hydrogel performance in the viscoelastic area under the strain sweep at a frequency of 6.28 rad/s should be further ensured.

**Figure 5:**
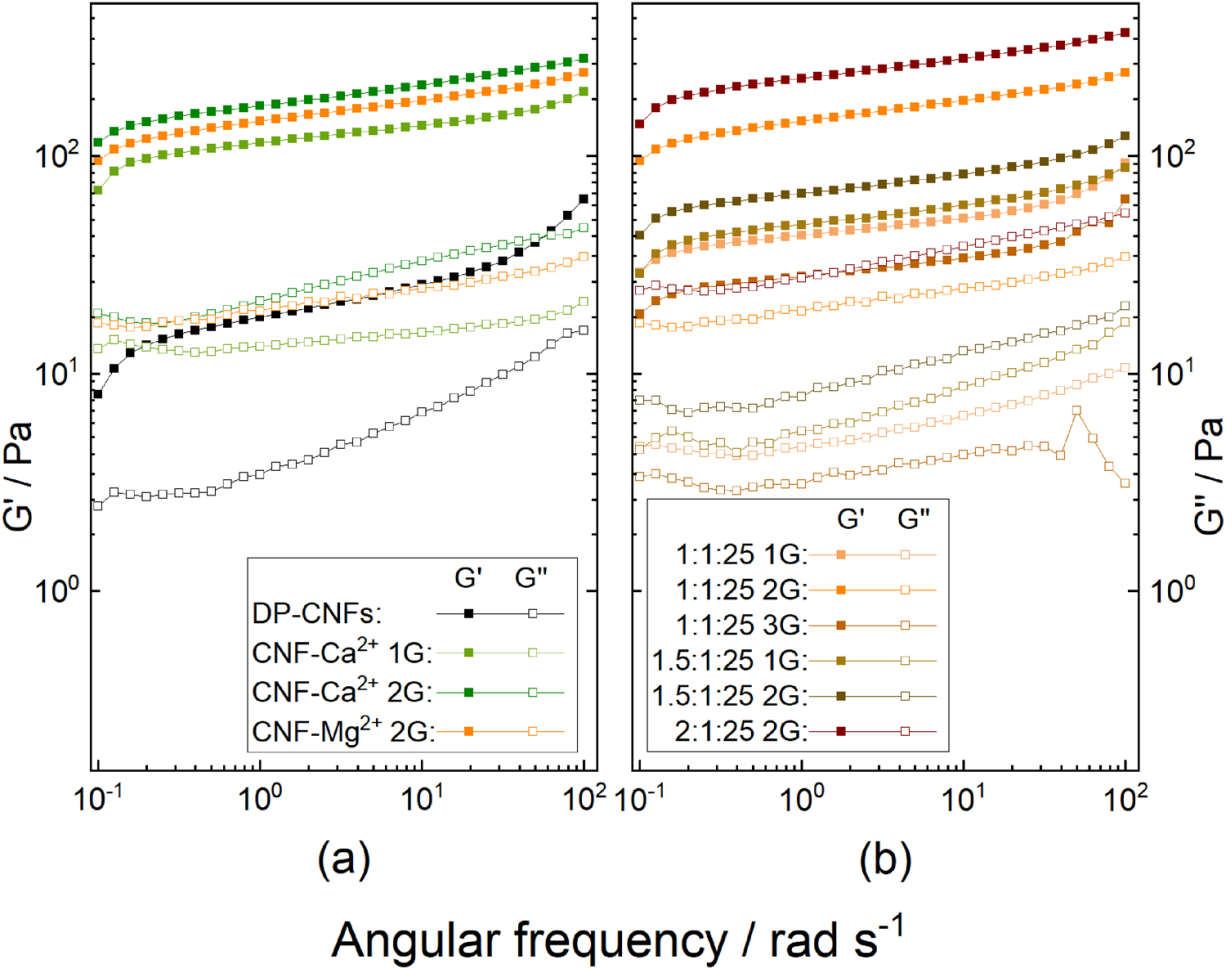
Viscoelastic properties of the deprotonated TOCNF dispersion and the hydrogels prepared from various divalent cations concentrations and centrifuging intensity: (**a**) hydrogels with defined ratio of M^2+^:TOCNF:H_2_O = 1:1:25 centrifuged with 1 × G and 2 × G, (**b**) TOCNF–Mg^2+^ with various Mg^2+^ concentrations and centrifuging intensities. Storage modulus (G′) and loss modulus (G″) are symbolized with filled and open symbols, respectively.

The G′ value of TOCNF–Ca^2+^, at an M^n+^:TOCNF:H_2_O ratio of 1:1:25, is one order of magnitude higher than G′ for the original deprotonated TOCNF dispersion, indicating a better elasticity of the hydrogels due to the incorporation of the metal cations (cross-linking). The impact of centrifuging intensity on hydrogels was further studied (Fig. 5). An increase of the centrifugation force increases the viscoelasticity of the hydrogels to a certain extent. The centrifugation effect on the TOCNF–Mg^2+^ hydrogel (0.92 to 1.39% solid content) is more significant than on the TOCNF–Ca^2+^ hydrogel (solid content remains constant).

The viscoelastic properties of the TOCNF–Mg^2+^ after intensive centrifuging, were in the similar range as of the optimal TOCNF–Ca^2+^ and also had a comparable good 3D printing performance as of the TOCNF–Ca^2+^.

## Conclusion and outlook

In this study, CNF-based hydrogel inks for 3D printing were prepared from TEMPO-oxidized CNF (TOCNF) with a solid content of 2.64 wt% and a carboxylate content 1.94 mmol/g. Divalent and trivalent metal cations were introduced to cross-link the deprotonated TOCNFs to form the corresponding hydrogels. The chemical functional groups of the original CNF suspension, the TOCNFs and TOCNF–M^n+^ hydrogels were analyzed with FT-IR, which demonstrated a better interaction between carboxylate anions and the divalent cations Ca^2+^and Mg^2+^ than with the trivalent cations Fe^3+^ and Al^3+^. The storage modulus (G′) and loss modulus (G″') of hydrogels incorporating with trivalent cations Fe^3+^ and Al^3+^ were significantly higher than thoughts with divalent cations Ca^2+^ and Mg^2+^. Hydrogel 3D printing performance was evaluated and showed that gel cross-linked with the divalent cations Ca^2+^ and Mg^2+^ had good printability and that the TOCNF–Ca^2+^ prepared with an M^n+^:TOCNF:H_2_O ratio of 1:1:25 under 1 × G centrifugation was the best. This gel had a solid content of 1.39 wt% and a storage modulus of G′ = 2 kPa. A comparable performance was achieved with TOCNF–Mg^2+^ at the same ratio by 2 × G centrifugation.

## Methods

### Materials

The dry cellulose source, elemental chlorine free (ECF) bleached softwood kraft pulp, was obtained from MERCER Stendal GmbH, Germany. The Northern bleached softwood kraft pulp was made from pine (30–60%) and spruce (40–70%), PFI-milled at 23 °C and 50% relative humidity. CNF was produced by passing the softwood kraft pulp through an M-110EH-30 Microfluidizer from Microfluidics. The grinding degree was analyzed with a Schopper-Riegler analyzer (KARL SCHRÖDER KG, Germany).

2,2,6,6-tetramethylpiperidine-1-oxyl (TEMPO, 98%), hydrochloric acid (37%, HCl), ethanol (96%), sodium hydroxide solution (0.5 M, NaOH), sodium bromide (99%, NaBr), iron(III) chloride (98%, FeCl_3_), aluminum nitrate nonahydrate (98%, Al(NO_3_)_3_), calcium chloride dihydrate (99%, CaCl_2_), and magnesium nitrate hexahydrate (99%, Mg(NO_3_)_2_) were purchased from Sigma-Aldrich and used as received. Sodium hypochlorite pentahydrate (available chlorine min. 40.0%) was purchased from TCI EUROPE N.V. and used as received. All syntheses were performed using MilliQ water. MilliQ water was purified via a PURELAB® Option-Q System, 0.055 µS cm^−1^.

### Characterization

The morphology of the different CNF gels was observed via ultra-high-resolution field emission scanning electron microscopy (FE-SEM) using a Hitachi S-4800. The dried CNF samples were mounted on sample supports using carbon tape and coated with a 5 nm layer of Pd/Pt with a Cressington 208HR under an inert atmosphere.

Attenuated Total Reflection Fourier Transform Infrared Spectroscopy (ATR-FTIR) was performed using a Bruker Vector 33 spectrometer. Measurements were performed by accumulating 256 scans in the spectral region of 4000–550 cm^−1^ with a spectral resolution of 2 cm^−1^.

Rheological tests were carried out with a TA Instrument AR 2000ex and the Advantage Software v5.8.2. Rheological tests were carried out with a 40 mm parallel-plate configuration and 1000 μm gap distance. The cation-cross-linked hydrogel was distributed onto the bottom plate. The frequency sweep was set up between 0.1 and 100 rad s^−1^ and a strain sweep was performed at an angular frequency of 6.28 rad s^−1^ to ensure the measurements were made in the linear viscoelastic region.

The conductivity titration was performed with a 721 NET Titrino from Metrohm. For purification and concentration, a centrifuge (Sorvall LYNX 6000) from Thermo SCIENTIFIC was used.

### Production of cellulose nanofibrils

CNF was produced via microfluidic treatment similar to previously described processes^8^. In a typical procedure 10 g dry cellulose pulp was suspended in 200 mL water and grinded until a degree of grinding of 75–80°SR (SR: Schopper-Riegler degrees, determined using SCHOPPER-RIEGLER method (DIN EN ISO 5267-1).) was reached. A Microfluidizer (M-110EH-30 Microfluidics) was used to disintegrate cellulose fibers into CNFs. The fiber suspension firstly passed through two z-shaped channels of 400 μm and 200 μm diameter under high pressure (15,000 Psi). This operation was repeated two times. Then, the fiber suspension passed through two thinner chambers with orifice widths of 200 μm and 100 μm successively under the pressure of 25,000 Psi. This operation was repeated four times. The CNF suspension was then concentrated by centrifugation, resulting in a 2.0 wt% CNF aqueous gel.

### TEMPO-mediated oxidation of the CNF

TEMPO-mediated oxidized CNF (TOCNF) was obtained by TEMPO-mediated oxidation in water at pH 10 as described previously^35^. In a typical synthesis, 500 mL CNF suspension (0.58 wt%) was added to a 100 mL solution of TEMPO (0.05 g, 0.32 mmol) and NaBr (0.3 g, 2.9 mmol). NaClO·5H_2_O (4.9 g, 66 mmol) was then added to initiate the reaction. The mixture was kept at room temperature and the pH was maintained to a value of 10 through the addition of 0.5 M NaOH solution over a period of 5 h. After 5 h, no further pH variation was observed, indicating the end of the reaction. The reaction was quenched by adding 15 mL ethanol. HCl solution (37 wt%) was then added to adjust the pH to 4. The suspension was concentrated by centrifugation (20,000×*g* for 45 min) yielding a solid content of 2.64 wt%.

A conductivity titration was performed to determine the carboxylate content^36^ of the TOCNF by titration with 0.05 M NaOH standard solution. A carboxylate group content of 1.94 mmol/g was measured. If compared to other already published articles, the carboxylate group contents of our TOCNF is higher, as other carboxylate group contents are more in the range of 1.0–1.5 mmol/g^24,33,37–40^. This high carboxylate group content has two mayor reasons. First of all, the cellulose was already fibrillated prior the oxidation process. This leads to a better accessibility for the oxidizing agent to the cellulose fibril compared to a procedure were the oxidation is part of the fibrillating process. More important is the fact, that a freshly made NaClO solution from a solid NaClO·5H_2_O source was used. In most other published procedures an already solved solution of NaClO in water is used and this solution will degrade over time and depending on the age of this solution the concentration will be lower.

### Preparation of cation-cross-linked TOCNF hydrogels

CNF hydrogels were produced through the addition of various metal cation solutions to cross-link the TOCNF in suspension. Before the addition of metal cation solutions, the pH of the TOCNF suspension was adjusted to 6 with a 0.5 M NaOH solution. The corresponding amount of the metal cation solution (50 mM, FeCl_3_, Al(NO_3_)_3_, CaCl_2_, or Mg(NO_3_)_2_) was added dropwise into the TOCNF suspensions. After 12 h, the hydrogels were collected through centrifugation for 20 min at defined g-force values of 4430×*g*, 8860×*g* or 13,290×*g*, which were redefined in this study as 1 × G, 2 × G, 3 × G respectively. The impact of centrifugation at different g-force values on hydrogel performance for 3D printing was also investigated. The hydrogels were characterized through ATR-FTIR spectroscopy and rheology measurements.

### 3D printing of hydrogels

A cube model of 10 × 10 × 5 mm was designed and 3D printed by pneumatic extrusion. 3D printing was performed with an INKREDIBLE 3D printer from CELLINK. The cubes were 3D printed using two different conical nozzles diameters (0.84 mm or 0.58 mm). The weights of the 3D printed cubes were measured before and after the drying process to determine the solid content of the hydrogels. The 3D printed cubes were according to their performances dried either in an oven overnight at 60 °C or through freeze-drying.

The 3D printed structures were characterized through visual inspection, ATR-FTIR spectroscopy and SEM.
